# Ovine gastrointestinal parasite burden and the impact of strategic anthelmintic treatment in community-based breeding sites in Ethiopia

**DOI:** 10.3389/fvets.2023.1094672

**Published:** 2023-03-21

**Authors:** Wassie Molla, Mesfin Mekonnen Moliso, Solomon Gizaw, Tesfalem Nane, Asrat Arke, Firdawok Ayele, Theodore Knight-Jones

**Affiliations:** ^1^Veterinary Epidemiology and Public Health, College of Veterinary Medicine and Animal Sciences, University of Gondar, Gondar, Ethiopia; ^2^Animal and Human Health Program, International Livestock Research Institute, Addis Ababa, Ethiopia; ^3^Areka Agricultural Research Center, Areka, Ethiopia; ^4^Bonga Agricultural Research Centre, Bonga, Ethiopia; ^5^Debre Berhan Agricultural Research Centre, Debre Berhan, Ethiopia

**Keywords:** anthelmintic, fecal egg count, *Fasciola*, gastrointestinal tract parasites, prevalence, sheep, strongylid, Ethiopia

## Abstract

**Introduction:**

In Ethiopia, small ruminants contribute significantly to livelihoods and food security but productivity is low with high disease burden and essential endoparasite control not widely practiced. The current study assessed worm burden and its control in three districts in Ethiopia.

**Methods:**

All sheep older than 3 months in nine villages were treated *en-masse* with albendazole and triclabendazole twice a year from 2018 to 2021. Treatments were administered under field conditions by animal health workers. Pre- and post-treatment data were assessed looking at fecal egg presence/absence and fecal egg per gram (EPG) count.

**Results:**

A total of 1,928 and 735 sheep were examined before and after deworming, respectively. Before treatment worms were detected in 54.4% (95% CI: 52.2–56.6) of sheep. Strongylid (30.4%) and *Fasciola* (18.2%) were the most frequently identified parasites. Animals living in wet mid-highland environments were more than 23 times more likely to have strongylid eggs in their feces and 5 times more likely to have eggs from any gastrointestinal tract (GIT) parasites detected, as compared to animals living in moist highland agro-ecology. Over the course of the 2018–2021 community intervention there was total elimination of animals with a high worm burden (EPG > 1,500), and elimination of a third of those with moderate infections. Mild infections remained, largely accounted for by strongylid, which remains at low levels in healthy sheep. However, there were signs of emerging drug resistance.

**Conclusion:**

Generally, sheep in smallholder systems in Ethiopia experience a needlessly large economic burden from GIT worms. Routine therapy reduces this burden but smart strategies are needed to limit the onset of drug resistance.

## Introduction

Small ruminants are of immense economic importance in Ethiopia contributing to the livelihoods of huge numbers of households. But disease and poor reproductive performance are the major impediments to small ruminant productivity in Ethiopia (1). Gastrointestinal parasite prevalence is very high (75.8%) and is a key cause of disease burden (2, 3). Under field conditions, most infections are usually mixed consisting of different species of nematodes, with growing lambs and peri-parturient ewes particularly susceptible (4).

Most of the economic losses caused by internal parasites are associated with production losses in terms of reduced milk and wool production, poor hair coat or fleece growth, loss of body weight, cost of prevention, cost of treatment and the loss of infected animals, but deaths also occur (5).

Routine anthelmintic treatment of small ruminants to control gastrointestinal tract (GIT) parasites is a recommended and accepted management practice to maintain and improve animal health and performance. However, the long-term sustainability of routine anthelmintic treatment is challenged by accelerating development of drug resistance in small ruminant parasites (6, 7). GIT parasites have been shown to develop resistance to anthelmintic drugs following repeated use of the same anthelmintic over time, particularly for certain parasite species and drug classes such as benzimidazoles (8). Over time, these resistant organisms will dominate the parasite population and jeopardize efforts to control worm burden (9).

One way to reduce anthelmintic resistance is to only treat animals within the flock that need treatment rather than treating the whole herd (10, 11). This requires close monitoring of parasite infection and an effective way of doing this is by performing fecal egg counts (FEC) on a sample of small ruminants and deciding if treatment is needed based on the results, withholding treatment if egg counts are low (9).

The fecal Egg Count Reduction Test (FECRT) is a way to measure the effectiveness of treatment, comparing pre and post-treatment egg counts. If the current anthelmintic is still effective, the number of parasite eggs per gram of feces should drop by at least 95% (12, 13). The use of fecal egg count in conjunction with FAMACHA^©^ scores (a correlate of anemia), presence of diarrhea, body condition scores, and overall animal health is recommended when deciding whether or not deworming is necessary (13).

The International Livestock Research Institute (ILRI) in Ethiopia has been implementing integrated animal health interventions since 2018 to overcome the challenges facing small ruminant producers in central, southern, and south western Ethiopia. The interventions include improved management, enhanced nutrition and the use of anthelmintics. Moreover, it includes strategic deworming. The objectives of this study were to: list the various types of pathogenic parasites and the factors favoring their occurrence in the study populations, assess the effect of strategic anthelmintic treatments in controlling GIT parasites, and evaluate the relationship of egg per gram with FAMACHA scores, body condition score, and packed cell volume.

## Materials and methods

### Description of the study areas

The study was carried out in three community-based breeding sites between the years 2018 and 2021. The breeding sites were located in Amhara (Menz district) and Southern Nations Nationalities People's (SNNP) (Adiyo and Doyogena districts) regional states of Ethiopia. Adiyo district has an altitude ranging from 500 to 3,500 m above sea level. The annual precipitation of the area is about 2,300 mm and the temperature varies from 3 to 36°C. Mixed crop-livestock production is the dominant farming system in the area (14). Doyogena is the other study district in SNNP region. Altitude of the district ranges from 1,900 to 2,800 m above sea level. Annual rainfall is 1,200–1,800 mm and the mean temperature varies from 10 to 18°C (15, 16). The third study district, Menz, has an altitude of 3,354 m above sea level. The district annually receives an average rainfall of 980 mm and its average temperature varies between 5 and 18°C (17). Sheep flocks in these study sites were managed by community-based breeding program members. The two intervention districts Adiyo and Doyogena have wet mid-highland agro-ecologies while Menz has a sub-moist highland agro-ecology.

### Situation of the intervention

A sheep gastrointestinal tract parasite control intervention program was initiated in community-based breeding villages; Keyafer, Sinamba-Boda, and Zeram from Menz district; Boka, Shena and Shuta from Adiyo district; and Ancha Sadicho, Hawara Arara, and Lemi Suticho from Doyogena district. Each community sheep breeding village shares communal grazing resources and watering points and but do mix with other village flocks. The GIT parasite control intervention employed deworming of all sheep more than 3 months old twice a year at the beginning of the long and short rainy seasons. Anthelminitics such as albendazole (Albentong 600, Congqing Fangtong Animal Pharmaceutical co. Ltd., China) and triclabendazole (Fascinex, Ciba-Geigy Ltd., Switzerland) were used for nematode parasites and liver flukes, respectively, following the recommended dose and routes of administration by manufacturers. Treatments were applied under field conditions by animal health workers and not as part of a controlled trial.

### Sampling and sample collection

A representative number of animals was selected randomly from each intervention village for physical examination and fecal and blood sampling before and after deworming. Pre- and post-treatment samples were not paired, with independent cross-sectional sampling at both pre- and post-treatment.

Fecal samples were collected from sheep for fecal egg count and parasite identification and speciation. The fecal samples were collected directly from the rectum. From each animal 5–10 g of fecal material was collected in a clean polythene bag containing 10% formalin as preservative. Blood samples (2 ml) were also collected from the jugular vein of the same animal into EDTA coated vacutainer tubes for determining the PCV.

Additional data collected from each animal comprised information on region, district, village, species, sex, age (young, 3–6 months and adult, 6 months and above; this was determined based on dentation and recorded data), fecal sampling, body condition score [BCS, scale 1–5 (18)], weight and examination date.

Sampling was done at time of treatment and 14 days later. Deworming and sampling were conducted twice per year for most sites, but in some sites was sometimes done once per year.

#### FAMACHA^©^ score

Sheep were individually examined to determine their FAMACHA^©^ score. The FAMACHA scores were determined by opening the lower eyelid of the sheep and comparing the color of the eye mucous membranes with a color chart bearing five categories of colors ranging from the normal, red through pink to practically white in severely anemic sheep (13, 19).

### Laboratory analysis

Laboratory methods such as floatation, sedimentation, and McMaster techniques were used to detect nematode and cestode eggs, trematode eggs, and to count the number of eggs or larvae per gram of feces and detect coccidia oocysts, respectively (20). Fecal samples that were positive for strongylid were subjected to egg counting. Based on fecal egg count, animals were categorized into mild infection (< 500 EPG), moderate infection (500–1,500 EPG) and high infection (>1,500 EPG) (21). However, attempts were not made to subcategorize the strongylid eggs to the contributing genera through differential coproculture or other methods.

### Fecal egg count reduction test

Fecal egg count reduction was computed by comparing the mean pre- and post-treatment EPG using the following formula (13, 22, 23).

FECR % = (1- mean post-deworming FEC/mean pre-deworming FEC) × 100

For this study, resistance was considered present in the worm population if the fecal egg count reduction percentage was found to be < 95% (22, 24).

### Statistical analysis

Data were cleaned and coded before statistical analysis using Stata 14. Descriptive statistics were produced. Prevalence of GIT parasitism was assessed based on the pre-intervention data. One-way analysis of variance (ANOVA) was employed to assess the mean EPG difference between age groups and districts.

Multilevel mixed effect logistic regression modeling taking district as a random effect was employed to assess the influence of variables (agro-ecology, age, sex, and BCS) on the detection of (1) strongylid, (2) *Fasciola*, and (3) any parasites regardless of species. After running univariable analysis, those variables with *P*-value < 0.25 and not collinear with each other were fitted in a multivariable model by a backward elimination procedure while checking for confounding. Pearson correlation test was also used to assess the relationship of EPG with FAMACHA^©^ score, PCV and BCS.

## Results

### Pre-intervention GIT parasite status

A total of 1,928 sheep from treated flocks were examined before deworming in the community-based intervention sites. Eggs were detected in 54.4% (95% CI: 52.2–56.6) of pre-deworming fecal samples (Table 1). The pre-deworming prevalence of GIT parasites in the three districts was 79.5, 73.8, and 35.1%, respectively, for Adiyo, Doyogena and Menz (Table 1) and it differed significantly (*P* < 0.001) between districts.

**Table 1:** The percentage of sheep with GIT parasite eggs of any species detected in their feces, by district, data from all years pooled.

**District**	**Age**	**Number examined**	**Number affected**	**Prevalence (95% CI)**	**P-value**
Adiyo	Adult	310	249	80.3 (75.9–84.6)	0.315
	Young	46	34	73.9 (61.2–86.6)	
	Subtotal	356	283	79.5 (75.3–83.7)	
Doyogena	Adult	393	279	71.0 (66.5–75.5)	0.018
	Young	161	130	80.8 (74.7–86.8)	
	Subtotal	554	409	73.8 (70.2–77.5)	
Menz	Adult	814	297	36.5 (33.2–39.8)	0.058
	Young	204	60	29.4 (23.2–35.7)	
	Subtotal	1,018	357	35.1 (32.1–38.0)	
Overall	Adult	1,517	825	54.4 (51.9–56. 9)	0.965
	Young	411	224	54.5 (49.7–59.3)	
	Total	1,928	1,049	54.4 (52.2–56.6)	

Strongylid (30.4%) and *Fasciola* (18.2%) were the most prevalent parasites recorded in this study. *Fasciola* prevalence was higher in Menz (25.5%) and Adiyo (24.6%) whereas strongylid prevalence was much higher in Doyogena (63.2%) and Adiyo (60.8%) districts (Table 2). When we see the relative frequency of the parasites, the most frequent parasite was strongylid (44.2%) followed by *Fasciola* (30.8%) and coccidia (10.6%).

**Table 2:** Pre-deworming prevalence of different parasite groups by district, year, and age.

**District**	**Year**	**Age category**	**No. sampled**	**No. of animal infected (%)**							
				***Fasciola***	***Paramphistomum***	**Strongylid**	***Trichuris***	***Moniezia***	**Coccidia**	**Others**	**Overall**
Adiyo	2018	Adult	33	13 (39.4)	0 (0.0)	23 (69.7)	0 (0.0)	0 (0.0)	3 (9.1)	3 (9.1)	27 (81.8)
		Young	14	5 (35.7)	0 (0.0)	11 (78.6)	0 (0.0)	2 (14.3)	0 (0.0)	1 (7.1)	12 (85.7)
	2019	Adult	83	24 (28.9)	2 (2.4)	-	1 (1.2)	-	-	0 (0.0)	76 (91.6)
		Young	6	2 (33.3)	0 (0.0)	-	0 (0.0)	-	-	0 (0.0)	5 (83.3)
	2020	Adult	117	30 (25.6)	0 (0.0)	-	7 (6.0)	0 (0.0)	0 (0.0)	0 (0.0)	98 (83.8)
		Young	7	1 (14.3)	0 (0.0)	-	0 (0.0)	0 (0.0)	0 (0.0)	0 (0.0)	5 (71.4)
	2021	Adult	77	11 (14.2)	-	42 (54.6)	0 (0.0)	4 (5.2)	-	0 (0.0)	48 (62.3)
		Young	19	1 (5.3)	-	11 (57.9)	1 (5.3)	0 (0.0)	-	0 (0.0)	12 (63.2)
Sub total				87 (24.6)		87 (60.8)					283 (79.5)
Doyogena	2018	Adult	174	0 (0.0)	0 (0.0)	79 (45.4)	-	17 (9.8)	37 (21.3)	0 (0.0)	104 (59.8)
		Young	100	0 (0.0)	0 (0.0)	53 (53.0)	-	17 (17.0)	42 (42.0)	0 (0.0)	79 (79.0)
	2019	Adult	24	0 (0.0)	-	15 (62.5)	-	6 (25.0)	5 (20.8)	0 (0.0)	18 (75.0)
		Young	13	0 (0.0)	-	9 (69.2)	-	2 (15.4)	6 (46.2)	0 (0.0)	12 (92.3)
	2020	Adult	95	3 (3.2)	-	95 (100)	4 (4.2)	6 (6.3)	16(16.8)	-	95 (100)
		Young	35	0 (0.0)	-	35 (100)	0 (0.0)	0 (0.0)	9 (25.7)	-	35 (100)
	2021	Adult	100	0 (0.0)	-	60 (60.0)	-	6 (6.0)	1 (1.0)	0 (0.0)	62 (62.0)
		Young	13	0 (0.0)	-	4 (30.8)	-	0 (0.0)	1 (7.7)	0 (0.0)	4 (30.8)
Sub total				3 (0.5)		350 (63.2)					409 (73.8)
Menz	2018	Adult	587	159 (27.1)	26 (4.4)	46 (7.8)	3 (0.5)	15 (2.6)	0 (0.0)	5 (0.9)	217 (37.0)
		Young	151	44 (29.1)	4 (2.6)	3 (2.0)	0 (0.0)	5 (3.3)	0 (0.0)	0 (0.0)	50 33.1)
	2019	Adult	67	11 (16.4)	2 (3.0)	4 (6.0)	0 (0.0)	0 (0.0)	0 (0.0)	0 (0.0)	17 (25.4)
		Young	13	0 (0.0)	0 (0.0)	0 (0.0)	0 (0.0)	0 (0.0)	0 (0.0)	0 (0.0)	0 (0.0)
	2020	Adult	74	0 (0.0)	0 (0.0)	0 (0.0)	-	-	-	21(28.4)	21 (28.4)
		Young	6	0 (0.0)	0 (0.0)	0 (0.0)	-	-	-	1 (16.7)	1 (16.7)
	2021	Adult	86	37 (43.0)	2 (2.3)	10 (11.6)	-	-	-	-	42 (48.8)
		Young	34	9 (26.5)	1 (2.9)	1 (2.9)	-	-	-	-	9 (26.5)
Sub total				260 (25.5)		64 (6.7)					357 (35.1)
Total				350 (18.2)		501 (30.4)					1,049 (54.4)

At the beginning (2018) of the intervention 82.6, 12.9, and 4.5% of the animals were grouped into mild, moderate and high worm egg infection categories, respectively. By the end of the community intervention program (2021) a reduction in egg burden could be seen with mild infections increased 9% reflecting reductions in moderate and severe infections (Table 3). The mean FEC of strongylid eggs was 351.2 EPG. The highest mean value was measured in Doyogena district (364.1). Young animals had significantly higher EPG count than adults, with the highest EPG for young animals reported from Doyogena (501.9) (Table 4).

**Table 3:** Categorization of sheep in three infection levels based on egg burden at the first (2018) and final (2021) pre-deworming sampling of the intervention.

**Period of examination**	**Number (%) of sheep**			**Total number of sheep tested**
	**Mild infection (**<**500 EPG)**	**Moderate infection (500–1,500 EPG)**	**Heavy infection (**>**1,500 EPG)**	
First (2018)	352 (82.6%)	55 (12.9%)	19 (4.5%)	426
Final (2021)	301 (91.5%)	28 (8.5%)	0 (0.0%)	329

**Table 4:** Mean egg count, by age and district.

**District**	**Age**	**Mean EPG (95% CI)**	**Std. Err**	***df* and *F***	**P-value**
Adiyo	Adult	343.9 (303.6–384.1)	20.4	(1, 307) = 6.21	0.0132
	Young	190.6 (114.9–266.3)	37.1		
	Combined	328.0 (290.8–365.2)	18.9		
Doyogena	Adult	307.6 (249.0–366.2)	29.8	(1, 552) = 6.08	0.0140
	Young	501.9 (305.7–698.1)	99.4		
	Combined	364.1 (293.5–434.7)	35.9		
Over all	Adult	322.6 (284.5–360.7)	19.4	(1, 861) = 4.92	0.0268
	Young	450.2 (285.6–614.9)	83.5		
	Combined	351.2 (304.0–398.3)	24.0		

### Factors related to GIT parasite occurrence

The univariable mixed effect logistic regression analysis result for the occurrence of strongylid, *Fasciola* and detection of parasite eggs of any species are shown in Table 5. Animals living in the wet mid-highlands were more than 23 and 5 times more likely to be affected by strongylid and any species of parasites respectively, as compared to those in sub-moist highland agro-ecology, otherwise no associations were detected in the final multivariable model. The variance and intracluster correlation coefficient (ICC) for the final model was 4.31e^−32^ and 1.31e^−32^, respectively.

**Table 5:** Univariable category prevalence and mixed effect logistic regression analysis to screen the risk factors for the occurrence of *Fasciola*, strongylid, and any species of parasites taking district as random effect.

**Risk factor**	**Number of animals positive/No. sampled (%)**			**Odd ratio (95% CI)**		
	***Fasciola***	**Strongylid**	**Overall parasite**	***Fasciola***	**Strongylid**	**Overall parasite**
**Agroecology:**						
SubmoistHL	260/1,018 (25.5)	64/953 (6.7)	357/1,018 (35.1)	Ref	Ref	Ref
Wet MHL	90/908 (9.9)	437/697 (62.7)	692/910 (76.0)	0.1 (0.0–6.9)	23.4 (17.4–31.4)	5.9 (4.8–7.2)
**BSC:**						
Poor	117/695 (16.8)	211/627 (33.7)	396/696 (56.9)	Ref	Ref	Ref
Medium	159/929 (17.1)	200/761 (26.3)	483/930 (51.9)	0.77 (0.6–1.0)	0.9 (0.7–1.2)	0.8 (0.7–1.0)
Good	56/255 (22.0)	56/215 (26.1)	131/255 (51.4)	0.90 (0.6–1.3)	2.1 (1.3–3.3)	1.1 (0.8–1.5)
**Age:**						
Young	70/430 (16.3)	139/406 (34.2)	239/430 (55.6)	Ref	Ref	Ref
Adult	280/1,496 (18.7)	362/1,244 (29.1)	810/1,498 (54.1)	1.0 (0.7–1.3)	1.2 (0.9–1.6)	1.1 (0.8–1.3)
**Sex:**						
Female	271/1,420 (19.1)	336/1,178 (28.5)	761/1,422 (53.5)	Ref	Ref	Ref
Male	79/506 (15.6)	165/472 (35.0)	288/506 (56.9)	1.0 (0.8–1.4)	1.2 (0.9–1.6)	1.2 (1.0–1.5)

### Post-intervention GIT parasite status and intervention effectiveness

A total of 735 sheep from treated flocks were examined after deworming. Eggs were detected in 44.8% (95% CI: 40.0–49.7) of post-deworming fecal samples (Table 6), 9.61% less than pre-treatment (*P* < 0.001). When we compared the pre-deworming prevalence of specific parasites with their post-treatment prevalence, the prevalence of *Fasciola, Moniezia, Paramphistomum, Trichuris* and others decreased significantly (Table 6). The proportion of sheep infected with *Fasciola* declined from 18.2 to 4.2% over the period of deworming interventions. However, the post-deworming prevalence of strongylid did not significantly decrease (Table 6).

**Table 6:** Overall pre- and post-deworming GIT parasite prevalence.

**Parasite type**	**Pre-deworming**		**Post-deworming**		**Difference significance *P*-value**
	**Positive/No. sampled**	**Prevalence in % (95% CI)**	**Positive/No. sampled**	**Prevalence in % (95% CI)**	
*Fasciola*	350/1,926	18.2 (16.5–20.0)	17/404	4.2 (2.6–6.7)	0.000
*Paramphistomum*	35/1,415	2.5 (0.4–1.8)	0/171	0 (0.0)	0.038
Strongylid	501/1,650	30.4 (28.2–32.6)	68/228	29.8 (24.2–36.1)	0.868
*Trichuris*	16/1,304	1.2 (0.8–2.0)	0/356	0 (0.0)	0.036
*Moniezia*	83/1,728	4.8 (3.9–5.9)	2/404	0.5 (0.1–2.0)	0.000
Coccidia	122/1,632	7.5 (6.3–8.9)	26/308	8.4 (5.8–12.1)	0.558
Others	31/1,678	1.9 (1.3–2.6)	0/404	0 (0.0)	0.006
**Overall**	**1,049/1,928**	**54.4 (52.2–56.6)**	**181/404**	**44.8 (40.0–49.7)**	**0.000**

The pre- and post-deworming GIT parasite EPG count and percentage of fecal egg count reduction (FECR) by year and district are shown in Table 7. The pre-treatment mean EPG had a decreasing trend from 2018 to 2021 in all districts. FECR results in Adiyo were 89.8% in 2019, 90.1% in 2020, and 68.0% in 2021; in Doyogena 97.6% in 2018, 93.5% in 2019, and 100% in 2020 and 2021; and in Menz 98.6% in 2018 and 85.7% in 2019 (Table 7).

**Table 7:** Pre- and post-deworming ovine GIT parasite average EPG count and percentage of FECR by district and year.

**District**	**year**	**Pre-deworming average EPG**	**Post-deworming average EPG**	**Difference**	**% FECR**
Adiyo	2019	304.2	31.1	273.1	89.8
	2020	442.3	44.0	398.3	90.1
	2021	203.7	65.1	138.6	68.0
Doyogena	2018	425.0	10.4	414.6	97.6
	2019	413.5	27.0	386.5	93.5
	2020	575.5	0.0	575.5	100
	2021	137.2	0.0	137.2	100
Menz	2018	420.1	5.8	414.3	98.6
	2019	57.5	8.2	49.3	85.7

### The relationship of mean egg count with FAMACHA score, BCS, and PCV

**Figure 1:**
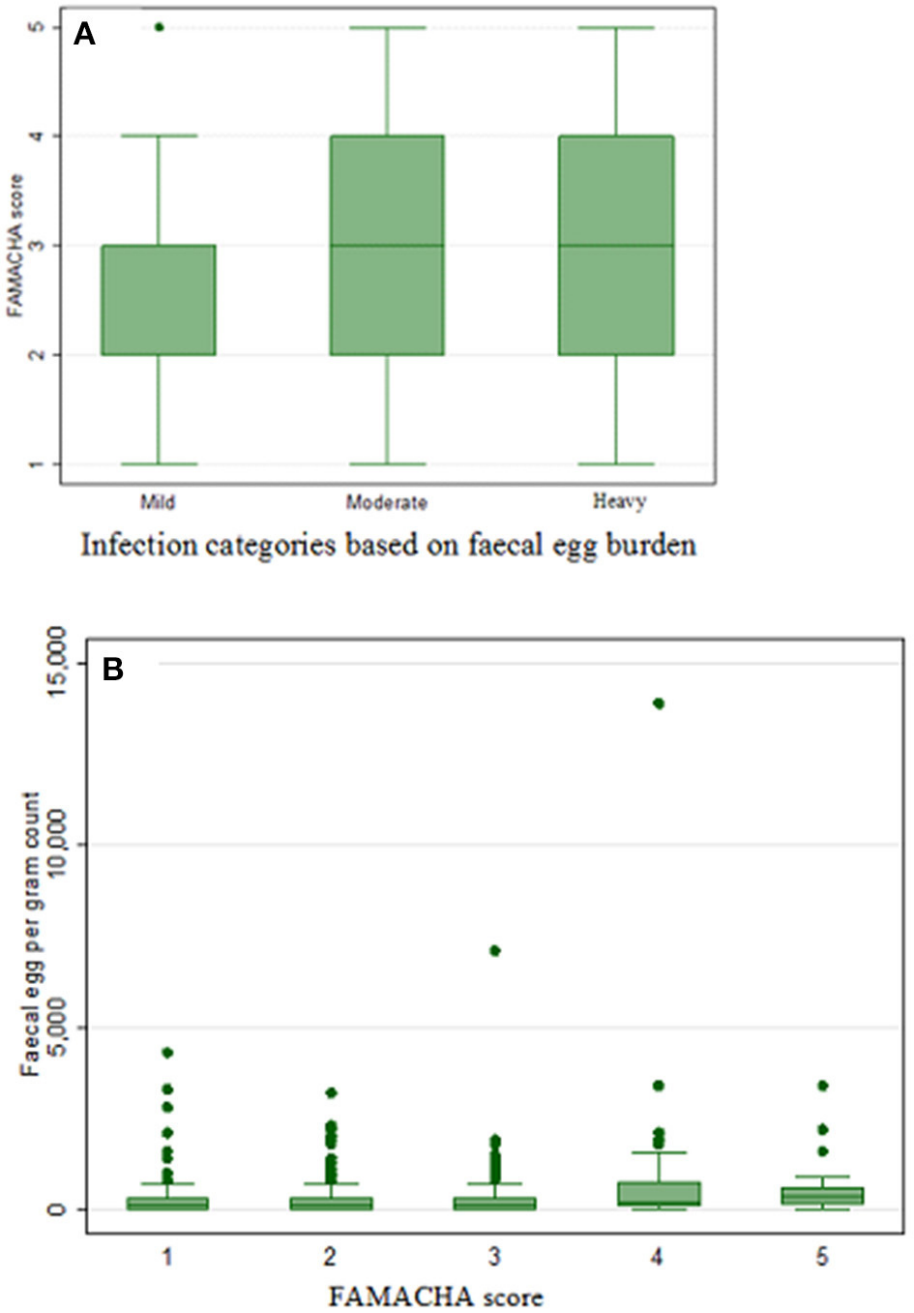
Box plot demonstrating the relationship between: **(A)** FAMACHA score and infection category, and **(B)** fecal egg count and FAMACHA score in sheep.

The FAMACHA scores and corresponding mean EPG values are shown in Table 8. The highest EPG count was recorded for FAMACHA score 4 (596.6) and 5 (635.0) (Table 8). The relationship of EPG and infection level to the different FAMACHA categories are demonstrated using box plot (Figure 1).

**Table 8:** FAMACHA score and the mean fecal egg count.

**FAMACHA score**	**No. of animal in each category**			**Total sample**	**Mean EPG (95% CI)**	**Standard error**
	**Mild infection**<**500 EPG**	**Moderate infection 500–1,500 EPG**	**Heavy infection** >**1,500 EPG**			
1	51	9	5	65	413.9 (212.0–615.7)	101.1
2	186	33	7	226	259.5 (198.9–320.2)	30.8
3	192	37	6	235	259.4 (187.2–331.5)	36.6
4	68	27	9	104	596.6 (316.7–876.5)	141.1
5	12	5	3	20	635.0 (238.4–1,031.6)	189.5

The relationship among EPG, FAMACHA scores, BCS, and PCV are indicated in Table 9. EPG and FAMACHA score correlated positively and significantly, whereas EPG correlated negatively but significantly with BCS and PCV (Table 9).

**Table 9:** Correlation among EPG, FAMACHA scores, BCS, and PCV.

**Parameter**	**Number of observation**	**Correlation coefficient**	**P-value**
EPG—FAMACHA	650	0.0958	0.0146
EPG—BCS	861	−0.0866	0.011
EPG—PCV	211	−0.3856	0.000

## Discussion

In Ethiopian, small ruminants kept in small holder systems are typically treated for endoparasites when clinical signs of disease appear. However, we show that over half the sheep in our study sites carried a subclinical worm burden. At the farm and the national scale this systematic lack of adequate parasite control causes massive economic loss through reduced production. To mitigate this problem, it is necessary to develop and implement a strategic GIT parasite control program that considers the epidemiology of the parasite and sets fixed time treatments that are aimed at decreasing pasture contamination (25).

Other studies have found an even higher prevalence [71% (2), 75.3% (26), and 86.6% (27)], although variation is expected with location, husbandry and climate. In our study pre-deworming prevalence varied between districts and this might be related to the difference in agro-ecology and husbandry practiced in the districts.

From the parasite groups identified and reported in this study, the most frequent and important are strongylid and *Fasciola*. These two pathogenic parasite groups accounted for 75.0% of parasites detected in the study areas. Animals that live in the wet mid-highlands (Doyogena and Adiyo) were more than 23 and 5 times more likely to be affected by strongylid and any species of parasites, respectively, as compared to those in sub-moist highland agro-ecology (Menz). This may be attributed to the wet and warm conditions, which favor the development and survival of the parasites (20), where parasite control is particularly important.

The 54.4% pre-deworming overall GIT parasite prevalence reported in the current study reduced to 44.8% post-treatment. This suggests a positive impact of the ongoing strategic GIT parasite control in the intervention sites with reduced contamination of pasture. As expected parasite prevalence reduced for most species, importantly *Fasciola*. However, the post-deworming prevalence of strongylid did not significantly decrease. However, the level of worm burden, reflected in worm egg counts, is more important than presence/absence of worm eggs, as healthy sheep should still have a low level worm infection and fecal egg counts reduced over the 2018–2021 course of the community intervention. Importantly there was total elimination of heavy infections from 4.5%, and moderate infections were reduced by a third to 8.5%. This reflects room for further improvement with better application as deworming was sometimes missed or erratically applied.

Most of the fecal egg count reduction results reported in this study were < 95%, indicating that the drugs used for the strategic deworming were not fully effective (22, 24), some were particularly low (68.0% in Adiyo in 2021). When egg count reductions fall below 90%, one can conclude that the proportion of resistant worms has probably increased to a level where another strategy should be considered (13). In the intervention sites, all sheep raised in the community breeding areas were dewormed indiscriminately without considering their EPG count, a practice which selects for resistance (9). It would be wise to employ selective and targeted use of anthelmintics to control GIT parasites. Performing fecal egg counts is helpful in monitoring infection and determining whether the animals need deworming or not.

Strongylid fecal egg count is a vital indicator of parasitic load and degree of pasture contamination. The parasite egg load result showed that the majority of animals examined had < 500 EPG and did not need treatment. Therefore, a targeted selective treatment approach would be advisable instead of mass deworming since it reduces the amount of drugs administered and decreases selection of resistant parasites. The higher mean EPG count reported in young animals than in adults is in line with what is expected since young animals are highly susceptible to GIT parasites as they have not yet developed immunity to the parasites, and should be prioritized for treatment.

High parasite load leads to weight loss and anemia resulting in a higher FAMACHA score, low PCV and BCS, reflected in our data. This result shows that the FAMACHA system may be used as an alternative to EPG in areas where veterinary laboratories do not exist, to identify animals that need deworming. In selecting based on clinical signs, rather than egg count the FAMACHA system can also help in selecting and breeding sheep best suited to the environment and better able to cope with GIT parasites (19). Furthermore, the observed positive correlation between FAMACHA and EPG, and the higher FAMACHA score and the low PCV value recorded in this study indicate the presence of blood sucking parasites in the stronglid group, mainly *Haemonchus* (19, 28) which are likely a major contributor to the health and production losses associated with parasitism in Ethiopia.

## Conclusion

High GIT worm burdens were detected in all sites, with *Fasciola* and strongylid being of particular concern, particularly in warm, moist areas. This exerts a large economic and welfare burden with morbidity and mortality leading to low productivity. Over the 4-year community intervention regular worming therapy was seen to eliminate heavy worm burdens, with BCS and live weight increasing with this reduction in worm burden. However, with the use of blanket worming therapy resistance to treatment was emerging in some sites. Alternative, targeted worming strategies should always be used to better manage this. The study demonstrated the need for well-designed worm control strategies in smallholder systems in Ethiopia to increase productivity whilst minimizing the development of resistance to essential parasite treatments. However, further work is required to quantify the economic benefit of such a program.

## Data availability statement

The raw data supporting the conclusions of this article will be made available by the authors, without undue reservation.

## Ethics statement

The animal study was reviewed and approved by International Livestock Research Institute Institutional Research Ethics Committee (ILRI IREC).

## Author contributions

SG and MM conceived and designed the study. MM, TN, AA, and FA undertaken the data collection and fecal examination. WM, MM, and TK-J analyzed, interpreted the data, and drafted the manuscript. All authors read, revised, and approved the final version of the manuscript for publication.
